# Phenological Response in the Trophic Levels to Climate Change in Korea

**DOI:** 10.3390/ijerph18031086

**Published:** 2021-01-26

**Authors:** Minkyung Kim, Sojeong Lee, Hakyung Lee, Sangdon Lee

**Affiliations:** Department of Environmental Science and Engineering, College of Engineering, Ewha Womans University, Seoul 120-750, Korea; enviecol@ewha.ac.kr (M.K.); lsojeong@naver.com (S.L.); hklee831@naver.com (H.L.)

**Keywords:** synchrony, global warming, species interaction, community, phenology

## Abstract

The response of the phenological events of individual species to climate change is not isolated, but is connected through interaction with other species at the same or adjacent trophic level. Using long-term phenological data observed since 1976 in Korea, whose temperature has risen more steeply than the average global temperature, this study conducted phenological analysis (differ-ences in the phenology of groups, differences in phenological shifts due to climate change, differ-ences in phenological sensitivity to climate by groups, and the change of phenological day differ-ences among interacting groups). The phenological shift of the producer group (plants) was found to be negative in all researched species, which means that it blooms quickly over the years. The regression slope of consumers (primary consumers and secondary consumers) was generally posi-tive which means that the phenological events of these species tended to be later during the study period. The inter-regional deviation of phenological events was not large for any plant except for plum tree and Black locust. In addition, regional variations in high trophic levels of secondary consumers tended to be greater than that of producers and primary consumers. Among the studied species, plum was the most sensitive to temperature, and when the temperature rose by 1 °C, the flowering time of plum decreased by 7.20 days. As a result of checking the day differences in the phenological events of the interacting species, the phenological events of species were reversed, and butterflies have appeared earlier than plum, Korean forsythia, and Korean rosebay since 1990. Using long-term data from Korea, this study investigated differences in phenological reactions among trophic groups. There is a possibility of a phenological mismatch between trophic groups in the future if global warming continues due to differences in sensitivity to climate and phenological shifts between trophic levels.

## 1. Introduction

Between 1880 and 2012, the average global surface temperature rose by 0.85 °C, while between 1912 and 2017, the average surface temperature in South Korea rose by about 1.8 °C [1]. Compared to the global and Korean temperature growth rates, it is clear that Korea has a higher temperature growth rate. Comparing the rate of change in Korea’s surface temperature over time, the trend of temperature increase appears to be stronger since 1973 [1].

The total number of species currently living on Earth is estimated at 8.7 million [2], and some species are more likely to become extinct due to global warming [3]. The risk of future extinction due to climate change is expected to accelerate as the global temperatures rises [4]. If future temperature changes are moderate, then about a quarter of species are predicted to disappear [5], which is a larger extinction rate than is expected due to habitat destruction [6]. Therefore, the possibility of future species extinction in Korea caused by climate change should be taken seriously.

Since plants and animals have a clear seasonality depending on the appropriate environment, their activities reflect seasonal patterns [7]. Changes in these phenological events (e.g., the flowering of plants and the emergence of butterflies, migratory birds, and frogs) are a means of determining the effects of climate change intuitively and quantitatively [8]. If long-term climate change causes a phenological event to occur at a different rate than the phenological events of the species interacting with it, it may lead to a mistiming of seasonal activity, or asynchrony [9]. Such phenological decoupling of food chains has serious consequences, including reduced biodiversity [10,11], and can thus threaten the function of ecosystems [12]. Nevertheless, a change in phenology or phenological rate does not mean asynchrony by itself [13], so it is necessary to consider the period of interaction between species.

Many studies have focused on phenological changes due to global warming and climate change at the level of individuals and single species [14]. The most common phenological response is the advancement of seasonal timing [15], and significant changes were observed within the taxonomic group or even among taxonomic groups, even among species that interact directly [16,17,18,19]. Phenological changes in species which interact with each other can lead to changes in synchrony along with the cascading community and ecosystem consequences [20]. It is necessary to identify phenological changes in multiple interacting species to investigate at the community level as well as at the species level, which must be accompanied by long-term data on sets of different species in both taxonomic and ecological aspects.

The differential phenological response to climate among species is predicted to interfere with trophic interactions, however data sets to test this prediction are rare [21]. During the flowering of plants that are producers, pollinators such as butterflies are needed. These insect pollinators are not only primary consumers, they also have predator–prey relations with birds and amphibians as secondary consumers. If these interacting organisms are no longer activated at the same time, a phenological mismatch could occur [22].

The timing of many phenological events results proximately from a complex interplay among an organism’s genes and several external environmental factors such as temperature, precipitation and photoperiod etc. [23]. Among them, the most influential climatic factor causing phenological changes is temperature [24,25]. Temperature has been used in many studies as a factor that explains phenological changes in consumer groups as well as in plant groups [12,26]. Within taxonomic groups, there can be substantial interspecific variation in phenological sensitivities, suggesting that there is likely to be variation due to temperature across taxonomic or trophic groups.

While numerous studies have been studying long-term trends, recent studies have shown that very long-term linear trends mask phenological shifts [27]. We had to take this into account. In consideration of the time of rapid warming in Korea, data for 40 years since 1975 were analyzed. Instead, the sensitivity to temperature before and after the warming period in Korea was compared. We also compared the day differences of phenological events between the interacting species.

Therefore, the study aims to evaluate the phenological changes of trophic groups in representative species inhabiting Korea, which has experienced rapid global warming. We conduct an analysis of phenological shifts resulting from climate change, along with differences in phenological variations by trophic levels and phenological sensitivities to climate by group and period. We also identified temporal trends about the day difference of phenological events between species that interact with each other.

## 2. Materials and Methods

### 2.1. Species Selection and Classification

Species from the phenological observations were selected by the Korea Meteorological Administration (KMA) to identify the biological seasonality of communities [28]. The protocols directed that a single individual plant for each species was observed close to each weather station for activity, and animal species were monitored at specific sites in the vicinity of each weather station where they were known to occur [27]. Phenological observations of plants and animals were conducted at the same locations every year in compliance with observation items, places, and methods [28].

This study analyzed 11 representative species of animals and plants that are widely distributed in South Korea. A total of seven plant species were investigated: plum tree (*Prunus mume*), Korean forsythia (*Forsythia koreana*), Korean rosebay (*Rhododendron mucronulatum*), cherry tree (*P. yedoensis*), peach tree (*P. persica*), pear tree (*Pyrus serotina*) and Black locust (*Robinia pseudoacacia*). One species of insect which is very common throughout Korea was studied, namely the cabbage butterfly (*Pieris rapae)*; one species of amphibian was studied, namely the black-spotted pond frog (*Rana nigromaculata*); and two species of bird were studied, namely the skylark (*Alauda arvensis*) and cuckoo (*Cuculus canorus*). Study species included interacting species, such as plants as producers and butterflies as pollinators, and prey and predators (insects and amphibians, insects and birds). The species also classified as: producers, primary consumers, and secondary consumers according to the status of organisms in the food chain in the ecosystem, i.e., the trophic levels (Table 1).

### 2.2. Data and Analysis

**Figure 1:**
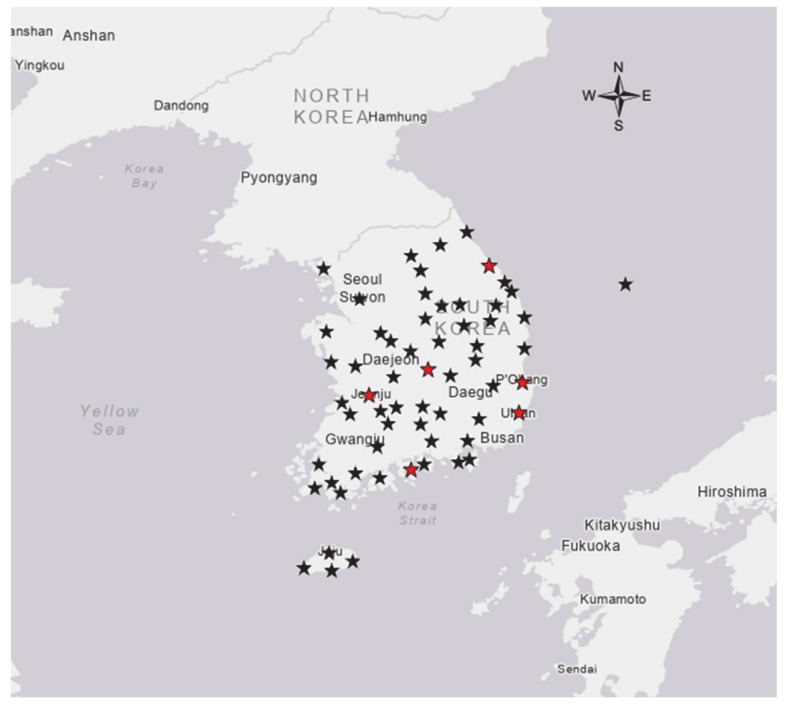
Locations of the 61 meteorological and phenological observation points in Korea that were used in this study. Among them, six sites marked by red stars used weather data and phenological data from 1936~1975 as well as 1976~2015.

The study used 21,089 phenological data of 40 years from 1976 to 2015 on 61 sites where 11 species of data exist (Figure 1). The phenological events used the First Flowering Date (FFD) for plants, the First Appearance Date (FAD) for insects and amphibians, and the First Singing Date (FSD) for birds. In particular, according to seasonal observation guidelines, FFD is recorded as a blooming day when three or more flowers bloom on any one branch of the tree [28]. The average phenological date of the species interacting with each other range from Julian days of 83.13~132.56, which was active during the spring season in South Korea (Table 1).

We conducted a regression analysis of the observed occurrence times of the phenological events at the 61 observation points for each species. We used the positive slope coefficient and negative slope coefficient value of the temporal trend relative to the year at each point. Significant regression coefficient values for 40 years were extracted to investigate phenological shifts in the groups at the trophic level.

We also used average temperature, which are typical weather factors that affect phenological events. The average temperature in the previous three months (February, March, April) were used in the timing of activities. In addition, to compare with the previous 40 years when temperature increases were lower, 1973 phenological data were used at the six points where 11 species of data existed between 1936 and 1975. We analyzed the regression of phenological events and temperature, and examined the coefficient of slope and R^2^.

Finally, we identified temporal trends about the day difference of phenological events between species that interact with each other. Therefore, we checked the phenological day differences between the seven species as producers and the cabbage butterfly as the pollinators, respectively, and checked the phenological day differences between the cabbage butterfly as prey and three species as the secondary consumers, respectively. We also conducted the temporal trend analysis for two different time periods, 1936~1975 and 1976~2015, in order to compare the changes in day differences between interacting species under different warming conditions. Hence we analyzed annual trends by calculating the day difference of interacting relationships at 61 sites during 1976~2015, and at six sites during 1936~1975 by site levels.

## 3. Results

### 3.1. Phenological Shift of Trophic Levels

We performed a regression analysis of the phenological events at the 61 observation points for each of the 11 species by year to examine the variations in the phenological trends for each of the species (Table 2). The species at each observation point were divided into two groups based on the slope values from regression analysis. A positive slope value for a species means that its phenological event became delayed during the observation period, while a negative slope value means that its event became advanced. A number of positive and negative slope coefficient values were resulted, and the number of statistically significant values (*p* < 0.05) is given in parentheses in Table 2.

For the seven plant species, which are producers, there were more stations where phenological events were advanced than stations where they were delayed, and the phenological events which were advanced had more statistically significant values. For the pollinator and primary consumer, cabbage butterflies, there were more stations where the phenological event was delayed, and the stations where the event was delayed often had statistically significant values. The phenological events of amphibians and birds, which are secondary consumers, were also delayed at more stations than they were advanced and often had statistically significant values in areas where they were delayed.

**Figure 2:**
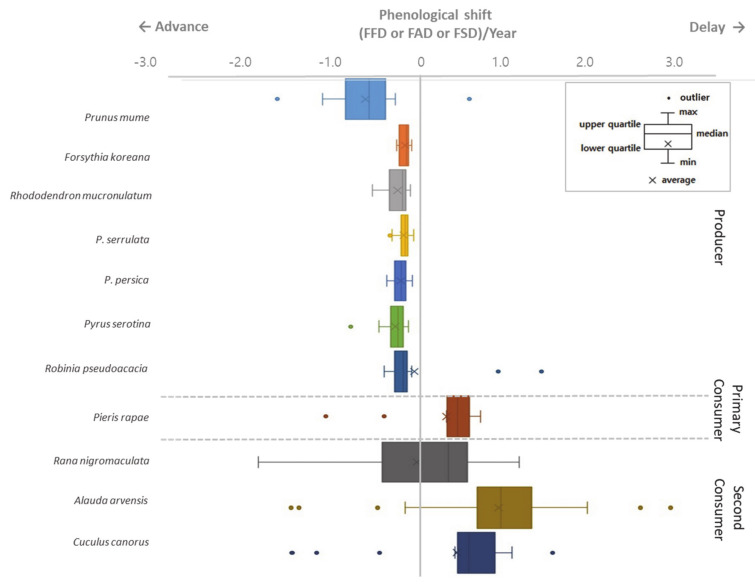
Phenological shift during 1976~2015 for each species with trophic structure of producer and primary and secondary consumers. The phenological shift represents the range of significant coefficient values for each species. The meaning of the boxplot is explained in the picture. (FFD = First Flowering Date, FAD = First Appearance Date, FSD = First Singing Date).

**Figure 3:**
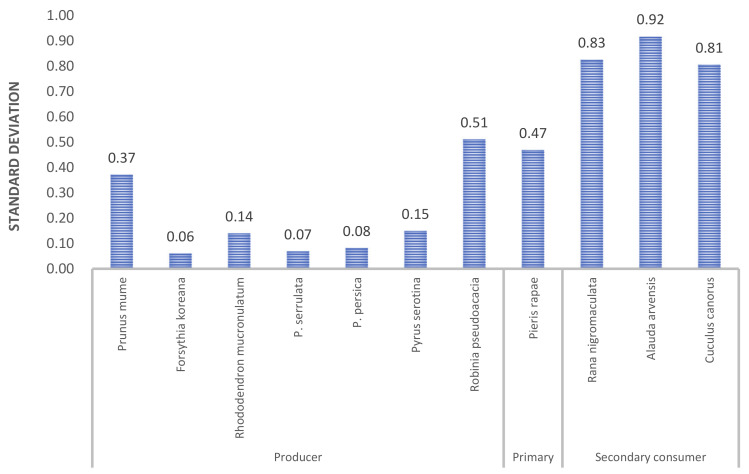
Standard deviation of significant coefficient values for each species.

Variation of statistically temporal trends of phenological dates of all species across 61 sites (Figure 2). In the y-axis, the species are arranged according to the order of phenological activity and trophic level. The phenological shift of all the studied plants is negative, that is, their phenological events occur progressively earlier during the study period. In particular, plum, whose phenological event occurs earliest, had the largest absolute value of phenological shift, meaning that its phenological event advanced the most during the study period. The regression slope of consumers (primary and secondary consumers) generally had positive values, meaning that the phenological events of these species tended to be delayed during the study period. Standard deviation of slope values can support regional variation here. Small standard deviation indicates small regional variation. The inter-regional deviation of phenological events was not large for any plant except for plum tree and Black locust (Figure 3). In addition, regional variations in high trophic levels of secondary consumers tended to be greater than that of producers and primary consumers.

### 3.2. Phenological Temperature Sensitivity

In relation to phenological events and climatic factors, all of the species tended to be earlier in activity as temperatures increased (Table 3). In the order of producers, primary consumers, and secondary consumers, were generally sensitive to temperature changes. Especially, the results show that all of the studied plant species and cabbage butterfly are significantly sensitive to temperature changes, although the degree of sensitivity differs from one species to another. Among the studied species, the plum tree was most sensitive to temperature both in 1976~2015 and 1936~1975; when the temperature rose by 1 °C, the flowering time of plums decreased by 7.2 and 5.83 days in relation to this.

In comparison by periods, except for Korean forsythia and Korean rosebay, 5 species of plants and cabbage butterfly were more sensitive to temperature during the last 40 years. Especially, for all species except secondary consumers, the R-square value of regression analysis with temperatures for 1976~2015 was higher than that for 1935~1975.

### 3.3. Change of Day Difference between Interacting Species

**Figure 4:**
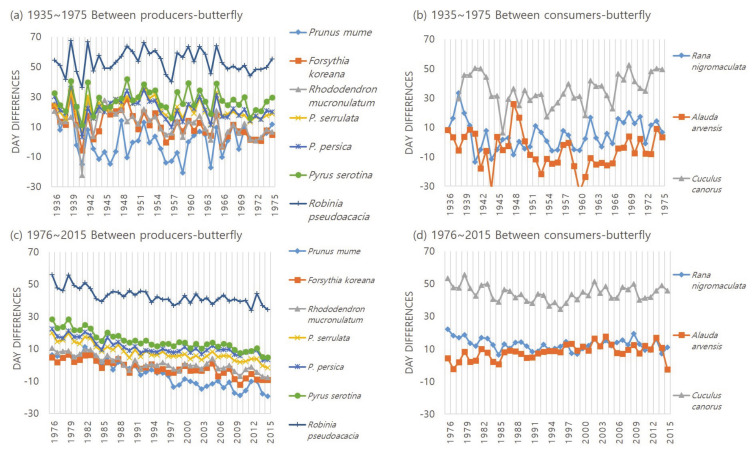
Variation in the day difference of phenological events between interacting species. The average day differences is calculated by subtracting the phenological date of the primary consumer, the butterfly, from the average phenological date of each species. Producers include 7 plant species to be studied, and consumers include 3 animal species of secondary consumer.

To examine the changes in biological season synchrony of interacting species, temporal trends were analyzed by calculating the day difference of the average phenological events between each trophic group (Figure 4). Increasing the number of day differences means that the dates of phenological events of each species are distant from each other, and the opposite is the decreasing day differences. The change of sign of day difference suggested the change of order of phenophase from the interacting species. During the 40-year period from 1935 to 1975, four of the seven plant–butterfly relations had a significant negative trend, while the other relations did not have a significant trends (Table 4). However, during the recent 40 years of 1976~2016, the day differences between all 7 plant species and butterflies has gradually significantly decreased. Adversely, the day difference between the skylark and the cabbage butterfly was significantly increasing.

## 4. Discussion

According to the Korea Climate Change Assessment Report 2020, which analyzed more than 1900 papers and reports, it is clear that since 1920 South Korea has experienced a higher rate of temperature increase compared to the global average, and since 1973 the trend of increasing temperature has been stronger [1]. In addition, there is a consensus that climate change is caused by human activities [29], and Korea is under high pressure in terms of ecosystem changes because of human intervention [30]. Using long-term phenological data observed at 61 locations in Korea, this study investigated phenological phenomena by dividing them into three ecological functional species groups (producers, primary consumers, and secondary consumers) that interact in the ecosystem. The slope of the regression equation was used to determine the phenological shift, with larger absolute slope values indicating greater variation in the occurrence time of the phenological event during the observation period.

First Flowering Dates of all plant species in this study showed advanced temporal shifts during 1976~2015. In particular, plum is the plant with the earliest FFD, with an average FFD of 83.13, and this species not only had a very large regional variation of phenological shift, but also had the most rapid advancement of its phenological event. This indicates that species with an early flowering date flower ever faster as the temperature increases. This is consistent with previous studies that showed that early-flowering plants are more sensitive to temperature increases [31,32,33]. If this trend continues in the future, there is a possibility that phenological asynchrony may intensify within the same trophic group and between each trophic group.

In the consumer group, which includes insects, amphibians, and birds, the phenological shift was positive, unlike the producer group (Figure 2 and Figure 3). This means that, in the consumer group, the phenological events were gradually delayed during the study period, and in general, regional variations were greater than those of plants. The striking differences between phenological changes in plants and animals in the region are potentially a reason for concern. It is possible that key plant–animal interactions, such as pollination, herbivory and also animal–habitat interactions could be disrupted by rapid changes in phenology [7,9]. Evidence for such temporal mismatches has been found in other, more intensively studied locations [34,35] but not in East Asia. Additionally, the spatial variation in phenological changes that we have observed could contribute to the potential for temporal mismatches, particularly for migratory species or species with large ranges. We believe that the potential for phenological mismatches is an area deserving of new research in East Asia and this study demonstrated the empirical evidence of ecological mismatches.

In other studies, phenological events of plants were found to have advanced rapidly during the past 40 years, whereas phenological events of flying birds have been found to show large variability [36].

In the situation where the producer’s phenological time is being advanced, the delay of the consumer’s phenological time indicates presumably an asynchrony implying differences by trophic group. For example, the average flowering time of plum becomes faster than that of the butterfly while the appearance of the cabbage butterfly (a pollinator) is gradually delayed (Table 1), and flowering dates of plants such as Korean forsythia and Korean rosebay are also becoming earlier (Figure 2). As a result, there is a possibility of problems in the fertilization and feeding process of plants by insects, and the ecosystem’s functions may collapse. To confirm this, the calculation of the day differences between the phenological events of the interacting species showed that the day difference between all species of plants and cabbage butterfly was significantly decreasing (Table 4). The day difference between plum, Korean forsythia, Korean rosebay and cabbage butterfly has been negative since 1990, and phenological events occurred before butterflies (Figure 4). That is, the change of sign of day difference suggested the change of order of phenophases in the interacting species.

Furthermore, by extracting and analyzing representative temperature factors that can cause phenological changes, it was found that all significant species have become more sensitive to temperature recently (Table 3). The slope of the regression equation for 1976~2015 of the studied species was (−7.20)~(−1.92), however the slope of the regression equation for 1936~1975 was (−5.83)~(−1.37), that is, the phenological time of the species responded more sensitively to the last 40 years than previous period. The all R^2^ value of the regression equation was higher for the recent 40 years (R^2^ = 0.13~0.83) than previous periods (R^2^ = 0.11~0.66). This indicates more explanatory power can be given to temperature in last 40 years to explain the variation of phenology than in the previous period. This is consistent with previous findings that, among climate factors, temperature should be a key factor for the change of the community [36].

Moreover, it was shown that the lower the trophic level, the more sensitive to temperature changes, and the higher the trophic level, the less it was affected by the temperature change during the recent 40 years (with significant results: 3.13 < |Slope| < 7.20 for the producer group, |Slope| = 1.92 for the primary and secondary consumer group). This indicates that the mechanisms affecting the phenological events of species differ by trophic group, which is consistent with previous studies that producers with lower food chains are more sensitive than consumers to temperature [37]. Plants that are producers respond directly to temperature changes, however, consumers are thought to be more affected by changes in ecological interactions caused by climate change (disruption of ecological network and phenological asynchrony) than by climate change itself [23,38].

Additionally, according to a prior study that described a delay in the observation period of swallows (*Hirundo rustica*), one of Korea’s spring birds, due to a decrease in the population [39], it is possible that the reason for the delay in the phenological date of consumer groups (including birds) in this study is a decrease in the population. Therefore, consideration of the size of the population will also be required in the future.

## 5. Conclusions

This study examined the differences in phenological responses between trophic groups using 40-year phenological data from 1976 to 2015 in Korea. It also compared this with the results of the previous 40 years from 1936 to 1975, when there was less warming. There is a possibility of mismatch between trophic groups in the future since there is a variation in sensitivity to climate and the phenological shift of trophic levels. Due to future global warming, large changes are expected to occur in the species and trophic levels, and consequently more diverse and integrated studies are needed. Our study suggests that it is necessary to develop climate change mitigation and adaptation strategies, food chain linkages, and biodiversity management plans.

## Figures and Tables

**Table 1:** List of species used in this study. Mean day of event refers to average dates of phenological event for each species during 40 years (1976~2015). And species are separated by trophic levels according to the status of organisms in the food chain in the ecosystem. (FFD = First Flowering Date, FAD = First Appearance Date, FSD = First Singing Date).

Classification	Species		Phenological Event	Mean Day of Event (Julian Day)	Trophic Level
	Common Name	Scientific Name			
Flora	Plum tree	*Prunus mume*	FFD	83.13 (SD = 19.12, *n* = 1898)	Producer (*n* = 7)
	Korean forsythia	*Forsythia koreana*	FFD	87.26 (SD = 8.32, *n* = 2065)	
	Korean rosebay	*Rhododendron mucronulatum*	FFD	89.92 (SD = 9.05, *n* = 2018)	
	Cherry tree	*P. yedoensis*	FFD	96.84 (SD = 7.97, *n* = 2067)	
	Peach tree	*P. persica*	FFD	99.87 (SD = 9.80, *n* = 2003)	
	Pear tree	*Pyrus serotina*	FFD	103.57 (SD = 8.89, *n* = 1890)	
	Black locust	*Robinia pseudoacacia*	FFD	131.53 (SD = 6.84, *n* = 1976)	
Insect	Cabbage butterfly	*Pieris rapae*	FAD	88.55 (SD = 12.54, *n* = 2021)	Primary consumer (*n* = 1)
Amphibian	Black-spotted pond frog	*Rana nigromaculata*	FAD	101.18 (SD = 12.97, *n* = 1850)	Secondary consumer (*n* = 3)
Avian	Skylark	*Alauda arvensis*	FSD	96.74 (SD = 19.45, *n* = 1514)	
	Cuckoo	*Cuculus canorus*	FSD	132.56 (SD = 13.98, *n* = 1787)	

**Table 2:** Temporal trends and positive and negative slope coefficient values for the regression of phenology vs. year at each location of 61 sites during 1976~2015. The number in parentheses indicates the number of statistically significant values (*p* < 0.05).

Tropic Level	Species	Trend to Earlier Spring Phenology (−)	Trend to Later Spring Phenology (+)
Producer	*Prunus mume*	55 (36)	6 (1)
	*Forsythia koreana*	53 (16)	8 (0)
	*Rhododendron mucronulatum*	46 (18)	15 (0)
	*P. yedoensis*	57 (32)	4 (0)
	*P. persica*	51 (16)	10 (0)
	*Pyrus serotine*	51 (24)	10 (0)
	*Robinia pseudoacacia*	47 (14)	14 (2)
Primary consumer	*Pieris rapae*	16 (3)	45 (16)
Secondary consumer	*Rana nigromaculata*	26 (10)	35 (13)
	*Alauda arvensis*	16 (4)	45 (29)
	*Cuculus canorus*	20 (3)	41 (14)

**Table 3:** The regression of phenology vs. average temperature (February–April) for each species during 1976~2015, 1936~1975. The bold numbers indicate the number of statistically significant values (*p* < 0.05).

Tropic Level	Species	1976~2015			1936~1975		
		Slope	*p*-Value	R^2^	Slope	*p*-Value	R^2^
Producer	*Prunus mume*	**−7.20**	**0.00**	**0.74**	**−5.83**	**0.00**	**0.22**
	*Forsythia koreana*	**−4.37**	**0.00**	**0.71**	**−5.27**	**0.00**	**0.53**
	*Rhododendron mucronulatum*	**−4.76**	**0.00**	**0.79**	**−5.13**	**0.00**	**0.40**
	*P. serrulata*	**−4.61**	**0.00**	**0.83**	**−3.60**	**0.00**	**0.66**
	*P. persica*	**−4.27**	**0.00**	**0.81**	**−4.18**	**0.00**	**0.51**
	*Pyrus serotina*	**−4.45**	**0.00**	**0.83**	**−2.53**	**0.00**	**0.28**
	*Robinia pseudoacacia*	**−3.13**	**0.00**	**0.62**	**−1.37**	**0.02**	**0.13**
Primary consumer	*Pieris rapae*	**−1.92**	**0.00**	**0.13**	**−2.82**	**0.04**	**0.11**
Secondary consumer	*Rana nigromaculata*	**−1.92**	**0.00**	**0.21**	−1.62	0.30	0.03
	*Alauda arvensis*	−0.08	0.95	0.00	−3.10	0.11	0.07
	*Cuculus canorus*	−0.16	0.79	0.00	−1.96	0.29	0.03

**Table 4:** The regression of day difference between interacting species by each sites during 1976~2015, 1936~1975. The bold numbers indicates the number of statistically significant values (*p* < 0.05).

Day Difference	Species	1976~2015			1936~1975		
		Slope	*p*-Value	R^2^	Slope	*p*-Value	R^2^
Producer-Primary consumer	*Prunus mume*	**−0.65**	**0.00**	**0.85**	−0.27	0.08	0.08
	*Forsythia koreana*	**−0.33**	**0.00**	**0.74**	**−0.54**	**0.00**	**0.46**
	*Rhododendron mucronulatum*	**−0.35**	**0.00**	**0.73**	**−0.34**	**0.01**	**0.16**
	*P. serrulata*	**−0.40**	**0.00**	**0.79**	**−0.33**	**0.00**	**0.30**
	*P. persica*	**−0.32**	**0.00**	**0.68**	**−0.28**	**0.00**	**0.27**
	*Pyrus serotina*	**−0.43**	**0.00**	**0.81**	−0.06	0.55	0.01
	*Robinia pseudoacacia*	**−0.31**	**0.00**	**0.55**	−0.09	0.40	0.02
Secondary consumer-Primary consumer	*Rana nigromaculata*	−0.08	0.13	0.06	0.09	0.43	0.02
	*Alauda arvensis*	**0.25**	**0.00**	**0.40**	−0.19	0.28	0.03
	*Cuculus canorus*	−0.07	0.32	0.03	0.16	0.32	0.03

## References

[1] Korea Meteorological Administration (2020). Korean Climate Change Assessment Report 2020.

[2] Mora C., Tittensor D.P., Adl S., Simpson A.G.B., Worm B. (2011). How many species are there on earth and in the ocean?. PLoS Biol..

[3] Lee S.D., Kwon S.-S. (2018). Carbon sequestration in the urban areas of Seoul with climate change: Implication for open innovation in environmental industry. J. Open Innov. Technol. Mark. Complex..

[4] Urban M.C. (2015). Accelerating extinction risk from climate change. Science.

[5] Thomas C.D., Cameron A., Green R.E., Bakkenes M., Beaumont L.J., Collingham Y.C., Erasmus B.F.N., De Siqueira M.F., Grainger A., Hannah L. (2004). Extinction risk from climate change. Nature.

[6] Pounds J.A., Puschendorf R. (2004). Ecology: Clouded futures. Nature.

[7] Visser M., Both C. (2005). Shifts in phenology due to global climate change: The need for a yardstick. Proc. R. Soc. B..

[8] Menzel A., Sparks T.H., Estrella N., Koch E., Aasa A., Ahas R., Alm-Kübler K., Bissolli P., Braslavská O., Briede A. (2006). European phenological response to climate change matches the warming pattern. Glob. Chang. Biol..

[9] Stenseth N.C., Mysterud A. (2002). Climate, changing phenology, and other life history traits: Nonlinearity and match-mismatch to the environment. Proc. Natl. Acad. Sci. USA.

[10] Visser M.E., Both C., Lambrechts M.M. (2004). Global climate change leads to mistimed avian reproduction. Adv. Ecol. Res..

[11] Thakur M.P. (2020). Climate warming and trophic mismatches in terrestrial ecosystems: The green–brown imbalance hypothesis. Biol. Lett..

[12] Thackeray S.J., Henrys P.A., Hemming D., Bell J.R., Botham M.S., Burthe S., Helaouet P., Johns D.G., Jones I.D., Leech D.I. (2016). Phenological sensitivity to climate across taxa and trophic levels. Nature.

[13] Kharouba H.M., Wolkovich E.M. (2020). Disconnects between ecological theory and data in phenological mismatch research. Nat. Clim. Chang..

[14] Walther G.-R. (2010). Community and ecosystem responses to recent climate change. Philos. Trans. R. Soc. B Biol. Sci..

[15] Ibáñez I., Primack R.B., Miller-Rushing A.J., Ellwood E., Higuchi H., Lee S.D., Kobori H., Silander J.A. (2010). Forecasting phenology under global warming. Philos. Trans. R. Soc. B Biol. Sci..

[16] Charmantier A., Mccleery R.H., Cole L.R., Perrins C., Kruuk L.E.B., Sheldon B.C. (2008). Adaptive phenotypic plasticity in response to climate change in a wild bird population. Science.

[17] Both C., Van Asch M., Bijlsma R.G., Burg A.B.V.D., Visser M.E. (2009). Climate change and unequal phenological changes across four trophic levels: Constraints or adaptations?. J. Anim. Ecol..

[18] Bauer Z., Trnka M., Bauerová J., Možný M., Štěpánek P., Bartošová L., Zalud Z. (2010). Changing climate and the phenological response of great tit and collared flycatcher populations in floodplain forest ecosystems in Central Europe. Int. J. Biometeorol..

[19] Thackeray S.J., Sparks T.H., Frederiksen M., Burthe S., Bacon P.J., Bell J.R., Botham M.S., Brereton T.M., Bright P.W., Carvalho L. (2010). Trophic level asynchrony in rates of phenological change for marine, freshwater and terrestrial environments. Glob. Chang. Biol..

[20] Kharouba H.M., Ehrlén J., Gelman A., Bolmgren K., Allen J.M., Travers S.E., Wolkovich E.M. (2018). Global shifts in the phenological synchrony of species interactions over recent decades. Proc. Natl. Acad. Sci. USA.

[21] Burthe S.J., Daunt F., Butler A., Elston D., Frederiksen M., Johns D., Newell M., Thackeray S., Wanless S. (2012). Phenological trends and trophic mismatch across multiple levels of a North Sea pelagic food web. Mar. Ecol. Prog. Ser..

[22] Miller-Rushing A.J., Høye T.T., Inouye D.W., Post E. (2010). The effects of phenological mismatches on demography. Philos. Trans. R. Soc. B Biol. Sci..

[23] Forrest J., Miller-Rushing A.J. (2010). Toward a synthetic understanding of the role of phenology in ecology and evolution. Philos. Trans. R. Soc. B.

[24] Lee S.D. (2017). Global warming leading to phenological responses in the process of urbanization, South Korea. Sustainability.

[25] Zhang J., Yi Q., Xing F., Tang C., Wang L., Ye W., Ng I.I., Chan T.I., Chen H., Liu D. (2018). Rapid shifts of peak flowering phenology in 12 species under the effects of extreme climate events in Macao. Sci. Rep..

[26] Kharouba H.M., Vellend M. (2015). Flowering time of butterfly nectar food plants is more sensitive to temperature than the timing of butterfly adult flight. J. Anim. Ecol..

[27] Fu Y., Piao S., Ciais P., Huang M., Menzel A., Peaucelle M., Peng S., Song Y., Vitasse Y., Zeng Z. (2016). Long-term linear trends mask phenological shifts. Int. J. Biometeorol..

[28] Korea Meteorological Administration (2016). Full Text of Seasonal Observation Guidelines.

[29] Cooke P. (2015). Green governance and green clusters: Regional & national policies for the climate change challenge of Central & Eastern Europe. J. Open Innov. Technol. Mark. Complex..

[30] Lee S.D. (2018). Wintering habitat use pattern of red-crowned cranes in the Korean demilitarized zone. J. Open Innov. Technol. Mark. Complex..

[31] Primack R.B., Ibanez I., Higuchi H., Lee S.D., Miller-Rushing A.J., Wilson A.M., Silander J.A. (2009). Spatial and inter-specific vaiability in phenological responses to warming temperatures. Biol. Conserv..

[32] Miller-Rushing A.J., Katsuki T., Primack R.B., Ishii Y., Lee S.D., Higuchi H. (2007). Impact of global warming on a group of related species and their hybrids: Cherry tree (Rosaceae) flowering at Mt. Takao, Japan. Am. J. Bot..

[33] Wolkovich E.M., Cook B.I., Allen J.M., Crimmins T.M., Betancourt J.L., Travers S.E., Pau S., Regetz J., Davies T.J., Kraft N.J.B. (2012). Warming experiments underpredict plant phenological responses to climate change. Nature.

[34] Inouye D.W., Barr B., Armitage K.B., Inouye B.D. (2000). Climate change is affecting altitudinal migrants and hibernating species. Proc. Natl. Acad. Sci. USA.

[35] Post E., Forchhammer M.C. (2007). Climate change reduces reproductive success of an Arctic herbivore through trophic mismatch. Philos. Trans. R. Soc. B Biol. Sci..

[36] Ovaskainen O., Skorokhodova S., Yakovleva M., Sukhov A., Kutenkov A., Kutenkova N., Shcherbakov A., Meyke E., Delgado M.D.M. (2013). Community-level phenological response to climate change. Proc. Natl. Acad. Sci. USA.

[37] Visser M.E. (2016). Interactions of climate change and species. Nature.

[38] Pérez-Ramos I.M., Cambrollé J., Hidalgo-Galvez M.D., Matías L., Montero-Ramírez A., Santolaya S., Godoy O. (2020). Phenological responses to climate change in communities of plants species with contrasting functional strategies. Environ. Exp. Bot..

[39] Lee S.-D., Ellwood E.R., Park S.-Y., Primack R.B. (2011). Late-arriving barn swallows linked to population declines. Biol. Conserv..

